# A Dynamic, Split-Luciferase-Based Mini-G Protein Sensor to Functionally Characterize Ligands at All Four Histamine Receptor Subtypes

**DOI:** 10.3390/ijms21228440

**Published:** 2020-11-10

**Authors:** Carina Höring, Ulla Seibel, Katharina Tropmann, Lukas Grätz, Denise Mönnich, Sebastian Pitzl, Günther Bernhardt, Steffen Pockes, Andrea Strasser

**Affiliations:** Institute of Pharmacy, Faculty of Chemistry and Pharmacy, University of Regensburg, 93040 Regensburg, Germany; ulla.seibel@ur.de (U.S.); katharina.tropmann@ur.de (K.T.); lukas.graetz@ur.de (L.G.); denise.moennich@ur.de (D.M.); sebastian.pitzl@ur.de (S.P.); guenther.bernhardt@ur.de (G.B.); steffen.pockes@ur.de (S.P.)

**Keywords:** histamine receptors, split-luciferase complementation (SLC), mini-G protein recruitment, G protein-coupled receptors (GPCRs), histamine receptor ligands, bioluminescence

## Abstract

In drug discovery, assays with proximal readout are of great importance to study target-specific effects of potential drug candidates. In the field of G protein-coupled receptors (GPCRs), the determination of GPCR-G protein interactions and G protein activation by means of radiolabeled GTP analogs ([^35^S]GTPγS, [γ-^32^P]GTP) has widely been used for this purpose. Since we were repeatedly faced with insufficient quality of radiolabeled nucleotides, there was a requirement to implement a novel proximal functional assay for the routine characterization of putative histamine receptor ligands. We applied the split-NanoLuc to the four histamine receptor subtypes (H_1_R, H_2_R, H_3_R, H_4_R) and recently engineered minimal G (mini-G) proteins. Using this method, the functional response upon receptor activation was monitored in real-time and the four mini-G sensors were evaluated by investigating selected standard (inverse) agonists and antagonists. All potencies and efficacies of the studied ligands were in concordance with literature data. Further, we demonstrated a significant positive correlation of the signal amplitude and the mini-G protein expression level in the case of the H_2_R, but not for the H_1_R or the H_3_R. The pEC_50_ values of histamine obtained under different mini-G expression levels were consistent. Moreover, we obtained excellent dynamic ranges (Z’ factor) and the signal spans were improved for all receptor subtypes in comparison to the previously performed [^35^S]GTPγS binding assay.

## 1. Introduction

G protein-coupled receptors (GPCRs) transduce external stimuli to intracellular events by the activation of heterotrimeric G proteins. Upon receptor activation, the heterotrimeric G protein binds to the receptor, which is followed by a GDP-GTP nucleotide exchange at the Gα subunit. The resulting conformational change of Gα promotes the uncoupling of the G protein from the receptor and the dissociation of the heterotrimer into a Gα monomer and a Gβγ dimer [1,2]. Both are then capable to modulate effector proteins inside the cell. Canonical GPCR-mediated signaling is determined by Gα, the subtypes of which target different membrane-bound effectors, such as phospholipase C [3,4] (PLC) and adenylyl cyclase [5,6] (AC). In drug discovery, GPCRs are the most studied drug targets and are addressed by more than 30% of approved drugs [7]. Fundamental criteria for successful drugs are a high binding affinity and potency at the target receptor, as well as a distinct pharmacological action ((full, partial, inverse) agonism, antagonism). The further downstream in the signaling cascade, the more pronounced the signal, irrespective of the ultimate cellular response. Thus, the characterization of the proximal functional response as a target-specific effect is desirable, particularly for lead-structure identification and bias analysis of compounds.

Classical methods have successfully focused on the key events of receptor-G protein interaction and G protein activation using radiolabeled GTP analogs ([^35^S]GTPγS [8,9,10], [γ-^32^P]GTP [11,12,13]). Unfortunately, we have repeatedly experienced insufficient quality with batches of commercially available radiolabeled GTP analogs. For this reason, compounded by economic considerations, such as the increased cost of radioactive waste disposal, it may be preferable to implement a different proximal functional assay, both for routine testing and detailed pharmacological studies of ligand-GPCR interaction. Non-radioactive labels of GTP analogs, such as europium [14], TAMRA, Cy3, and Cy5 [15], as well as the utilization of the commercial GTPase-Glo^TM^ technique [16], in which native GTP is converted to ATP, which is then involved in an enzyme reaction, allow for a fluorescent or bioluminescent readout. However, these methods are restricted to membrane preparations, cell homogenates or fixed cells [16,17]. Moreover, nucleotide exchange and GTP hydrolysis represent limiting steps according to the respective Gα subtype [18]. Modern FRET-/BRET-based G protein activation sensors monitoring the interaction of appropriate donor-acceptor pairs (GPCR and Gα/Gβγ [19], Gα and Gβγ [20] or Gβγ and a membrane anchor [21]) provide valuable insight into signaling kinetics and can visualize signal compartmentalization. However, for routine characterization of potential ligands, the application of these sensors is unfavorable due to the requirement for specialized equipment (e.g., multiple wavelength monitoring) and comprehensive expertise in performing the time-sensitive technique (millisecond timescale). Additionally, the spectral properties of the donor/acceptor pairs (intensity and spectral overlap of the excitation and emission wavelengths) can affect the signal amplitude [18]. This is an issue in case of weakly expressed GPCRs.

In 2017, a new class of minimal G protein chimeras (mini-G) was developed. All mini-G constructs are surrogates of the Gα_s_ subunit and comprise the following key features: minimization to the GTPase domain, a mutation that uncouples the binding to active state GPCRs from nucleotide exchange, and the deletion of the N-terminal membrane anchor as well as the Gβγ binding site. By replacing the α5 helix of the minimal Gα_s_ protein (mGs) with the respective sequence of other Gα subunits, mini-G proteins covering all major Gα families were derived and appropriate coupling specificities were demonstrated [22]. The application of BRET and split-luciferase complementation (SLC) techniques to GPCRs and mini-G proteins has created new G protein sensors that monitor functional responses in real-time [23,24,25,26,27]. Of particular note, the dynamic assay ranges benefited from the cytosolic nature of the mini-G proteins, as native, membrane-anchored G proteins produce high baseline values due to their closer proximity to membrane-bound GPCRs [23,24,25].

The aim of this study was to implement a modern, live cell-based assay to study the molecular signaling mechanisms of putative histamine receptor agonists and antagonists. Moreover, the method needed to provide a proximal readout with improved signal amplitudes, which was essential for the weakly expressed H_4_R [28]. For these purposes, the mini-G protein concept was considered suitable. We applied the split-NanoLuc technology [29] to all four histamine receptor subtypes (H_1_R, H_2_R, H_3_R and H_4_R) and the respective (chimeric) mini-G proteins mGsq, mGs and mGsi, where mGsq and mGsi represent chimeras of mGs with respective α5 helices of Gα_i_ and Gα_q_. Our study reports on the evaluation of mini-G sensors for the entire histamine receptor family, including functional characterization of standard histamine receptor ligands.

## 2. Results

### 2.1. Principle and Characteristics of the Mini-G Protein Recruitment Assay

**Figure 1:**
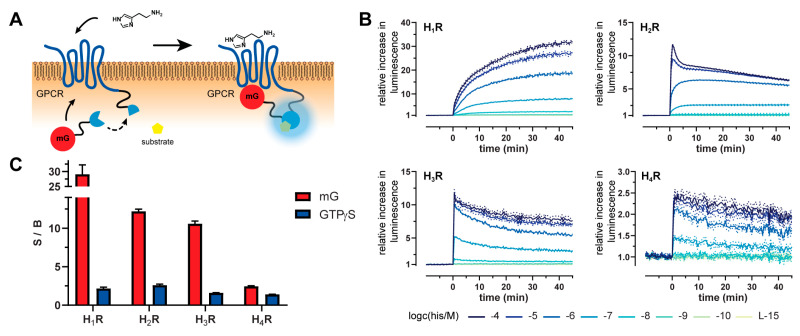
Principle of the mini-G protein recruitment assay and obtained signals at the H_1–4_R. (**A**) Scheme of the mini-G protein recruitment assay. The split-NanoLuc technology was applied to the H_1–4_R (C-terminus) and the mini-G proteins (mG; N-terminus). Upon receptor activation, the mini-G protein is recruited to the GPCR and the split-NanoLuc fragments form a functional enzyme leading to the oxidation of the substrate and thus luminescence signals in an agonist concentration-dependent manner. (**B**) Representative luminescence traces of the mini-G protein recruitment of mGsq to H_1_R, mGs to H_2_R and mGsi to H_3_R and H_4_R. Baseline and inter-well corrected luminescence traces of histamine at various concentrations and the assay medium Leibovitz’s L-15 (L-15) as negative control are plotted. (**C**) Plotted signal-to-background ratios (S/Bs) were calculated from 100% and 0% values of the respective assays, representing top and bottom values of the concentration response curves. For the mini-G recruitment assay (mG), peak or plateau values of the response to 100 µM histamine (100%) and L-15 (0%) are displayed, whereas for the [^35^S]GTPγS binding assay (GTPγS) responses to 1 mM histamine for H_1,2_R or to 10 µM histamine for H_3,4_R (100%) and H_2_O (0%) were taken. Presented data are the means ± SEM of at least five independent experiments (*n* ≥ 5), each performed in triplicate.

To study the G protein signaling of histamine receptor ligands, we applied the split-NanoLuc technology [29] to the human histamine receptor subtypes H_1_, H_2_, H_3_ or H_4_ (NlucC) and the corresponding mini-G proteins mGsq, mGs and mGsi (NlucN) (Supplementary Figure S1). Upon receptor activation, the mini-G protein was recruited by the receptor leading to the formation of a functional NanoLuc (Figure 1A). Thus, agonist concentration-dependent luminescence signals were obtained in the presence of the substrate furimazine (Figure 1B). To investigate antagonists, the response of the reference agonist histamine at EC_80_ concentration (H_1_R: 10 µM, H_2–4_R: 1 µM) was measured after a pre-incubation period of the respective antagonists. In order to verify the histamine receptor expression, radioligand saturation binding experiments were performed, and adequate binding of [^3^H]mepyramine to the H_1_R co-expressed with mGsq, [^3^H]UR-DE257 to the H_2_R co-expressed with mGs and [^3^H]UR-PI294 to the H_3_R and H_4_R each co-expressed with mGsi were observed (Supplementary Figure S2 and Table S1).

### 2.2. Kinetics and Dynamic Ranges of Mini G Protein Recruitment 

The dynamic split-NanoLuc approach allows for monitoring the G protein response to a ligand in real-time, demonstrating the differences in kinetics for each receptor and mini-G protein combination upon histamine stimulation (Figure 1B). The mGsq recruitment to the H_1_R is comparatively slow, leading to a plateau, whereas the luminescence signals of the mGs and mGsi recruitment to the H_2_R, H_3_R and H_4_R reach very sharp maxima and then flatten gradually. As the deletion of the membrane anchor and the Gβγ binding site were key modifications in the development of the utilized mini-G proteins, we assume that these observed kinetics will differ to the behavior of endogenous heterotrimeric G proteins. Further, other properties of the test system, such as the split-luciferase complementation reaction and the protein expression levels of the receptors and mini-G proteins, could influence the kinetics. Nevertheless, tracing the mini-G protein recruitment upon receptor activation in real-time could unveil differences in receptor regulation (e.g., receptor desensitization and internalization) [30,31] and may also serve as a useful tool to supplement studies of ligand binding kinetics, such as association and dissociation rate constants (*k*_on/off_) and residence time [32,33].

Using the mini-G sensors, the signal amplitudes of the assay were improved for all four receptor subtypes compared to the [^35^S]GTPγS binding assay (Figure 1C). For uniform comparison of the signal-to-background (S/B) ratios, we also implemented the [^35^S]GTPγS binding assay for the H_1_R (Table S1, Supplementary Methods). Remarkably, in the case of the H_1_R, the S/B ratio was up to 29-fold higher in the mini-G protein recruitment assay than in the [^35^S]GTPγS binding assay (Figure 1C). Such favorable S/Bs are beneficial for the determination of agonist efficacies and will allow for a reduction of the agonist concentration when exploring antagonists. To evaluate the overall assay quality, we calculated the Z’ factor, a dimensionless figure of statistical effect size. Classically, the Z’ factor has been used in the validation process of HTS methods, as it numerically evaluates the dynamic range of an assay and its ability to identify biologically active molecules [34]. For all four receptor subtypes, we obtained a Z’ factor that was between 0.5 and 1.0 (H_1_R: 0.79 ± 0.07, H_2_R: 0.85 ± 0.03, H_3_R: 0.80 ± 0.04, H_4_R: 0.68 ± 0.05; Supplementary Figure S3) indicating a sufficient separation of maximal effect and baseline values. Consequently, the presented mini-G protein recruitment assays can be classified as excellent screening methods [34].

### 2.3. Mini-G Protein Recruitment-Based Investigation of Histamine Receptor Ligands with Diverse Pharmacological Profiles

**Figure 2:**
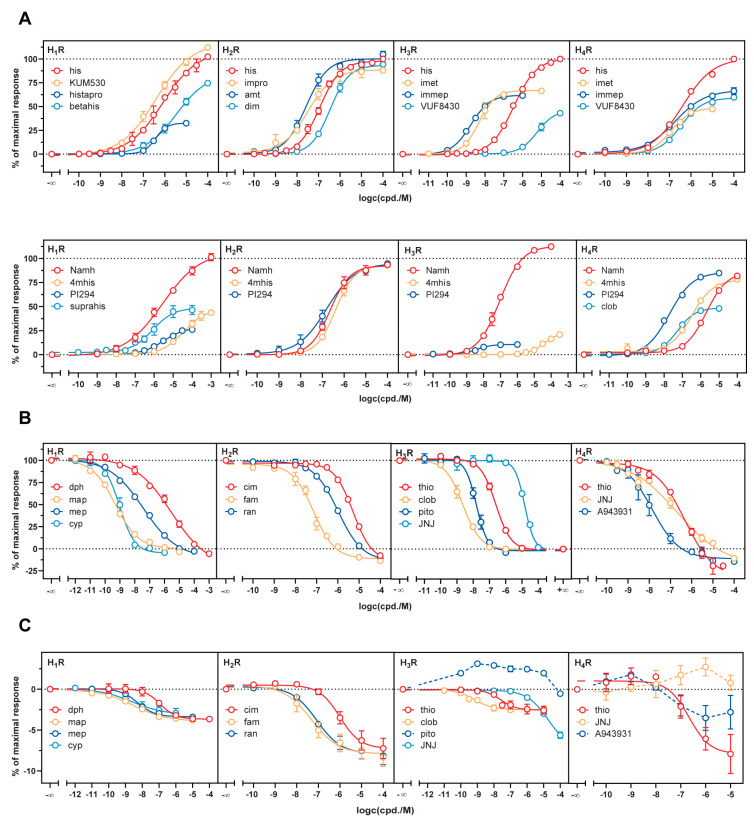
Concentration response curves obtained in the mini-G protein recruitment assay using agonists (**A**) in agonist mode, as well as antagonists in antagonist mode (**B**) and agonist mode (**C**). In agonist mode, the effect of the ligands themselves was tested, whereas experiments in antagonist mode were performed in the presence of the agonist histamine (H_1_R: 10 µM, H_2–4_R: 1 µM). HEK293T cells stably co-expressing a combination of either the H_1_R-NlucC/ NlucN-mGsq, H_2_R-NlucC/ NlucN-mGs, H_3_R-NlucC/ NlucN-mGsi or H_4_R-NlucC/ NlucN-mGsi were used. Data were normalized to L-15 as solvent control and to maximal responses elicited by 100 µM histamine in the case of agonists, 10 µM histamine for H_1_R antagonists or 1 µM histamine for H_2–4_R antagonists. Data represent means ± SEM from at least three independent experiments (*n* ≥ 3), each performed in triplicate.

To demonstrate the applicability of these novel assays for future drug research, we tested a set of standard ligands (Supplementary Figure S4), which are described as (inverse) agonists or antagonists. We experienced a broad range of potencies and efficacies for ligands at all four receptor subtypes (Figure 2) and the order of potencies of all studied agonists was in good agreement with literature data (Figure 2A, Table 1, Table 2, Table 3 and Table 4). However, as general observation, agonists probed at the H_3_R and the H_4_R displayed lower potencies (up to one magnitude) than in published [^35^S]GTPγS binding and steady-state GTPase activity assays (cf. Table 3 and Table 4). Likewise, this phenomenon was observed for agonists studied in NanoBRET binding assays using intact cells expressing either the H_3_R or the H_4_R, as well as for agonists investigated with a H_3_R conformational sensor [35,36,37]. This finding was proposed as a consequence of an altered GPCR- G protein-guanine nucleotide composition, and therefore a more transient formation of the ternary complex compared to cell membrane preparations or cell homogenates [17]. By testing a large set of agonists, we validated the mini-G protein recruitment approach to report on a multifaceted spectrum of pharmacological actions. Efficacies ranged from weak partial agonism, discovered for histaprodifen at the H_1_R (E_max_ = 33% ± 2.0) and UR-PI294 at the H_1_R (E_max_ = 29% ± 1.4) and H_3_R (E_max_ = 11% ± 1.1), to full agonism, demonstrated by e.g., *N*^α^-methylhistamine at the H_1_R (E_max_ = 99% ± 2.0), dimaprit at the H_2_R (E_max_ = 94% ± 2.6) and histamine (by definition: 100%) at all four receptor subtypes (Table 1, Table 2, Table 3 and Table 4). Strikingly, the efficacies of UR-KUM530 at the H_1_R (E_max_ = 112 ± 1.0) and *N*^α^-methylhistamine at the H_3_R (E_max_ = 111 ± 1.6) were significantly higher (α < 0.05) as those of the endogenous ligand histamine (Table 1 and Table 3), which is hypothesized as “superagonism” [38]. Similar results were previously observed for UR-KUM530 and were suggested to originate from a differing orientation in the binding pocket of the H_1_R compared to histamine [39,40]. In contrast, *N*^α^-methylhistamine has always been reported as a full agonist at the H_3_R [8].

Additionally, we extended the application of the mini-G sensor to the characterization of antagonists. The cells expressing the histamine receptors in combination with the respective mini-G proteins were pre-incubated with the antagonists and the response to the subsequently added agonist histamine was assessed. In this setting, standard antagonists exhibited expected p*K*_b_ values at all receptor subtypes (Figure 2B, Table 1, Table 2, Table 3 and Table 4). Only in the cases of the tricyclic H_1_R antagonists maprotiline (p*K*_b_ = 10.58 ± 0.11) and cyproheptadine (p*K*_b_ = 10.19 ± 0.10), we determined up to two magnitudes higher p*K*_b_ values than reported (Table 1). In the past, histamine receptors were reported to be constitutively active [41] in recombinant systems [42,43,44,45]. Investigation of the inverse agonistic potential of antagonists revealed that nearly all antagonists reduced the basal activity of the histamine receptors in the mini-G protein recruitment assay in a concentration-dependent manner (Figure 2C, Table 1, Table 2, Table 3 and Table 4). However, in our system the maximal inverse efficacies were small (H_1_R: −4%, H_2_R: −8%, H_3_R: −3%, H_4_R: −8% normalized to 100 µM histamine). Contrary to the literature, the constitutive activity of the Gα_i_-coupled receptors H_3_R and H_4_R was less pronounced [10,46]. However, as thioperamide demonstrated inverse agonism at the H_4_R, we confirmed JNJ7777120 and A943931 as neutral H_4_R antagonists.

**Table 1:** Potencies (pEC_50_/p*K*_b_) and efficacies (E_max_) of ligands at the H_1_R explored in the mini-G protein recruitment assay. Data represent means ± SEM of at least three independent experiments (*n* ≥ 3), each performed in triplicate. Statistical differences (*) of E_max_ > 100% was tested using a one-sample t-test (*n* = 5; α = 0.05). Functional data obtained from [S]GTPγS and steady-state GTPase assays and ligand binding affinities (p*K*_i_) determined in radioligand competition binding assays are included for comparison.

Compound	Mini G Protein Recruitment		GTPγS/GTPase ^‡^		Competition Binding
	pEC_50_/(p*K*_b_)	E_max_ [%]	pEC_50_/(p*K*_b_)	E_max_ [%]	p*K*_i_
his	6.16 ± 0.09	100	5.21 ± 0.06 ^a^ 6.92 ^‡,b^	100 ^a^ 100 ^‡,b^	5.62 ^h^
KUM530	6.41 ± 0.12	112 ± 1.0 *	6.22 ± 0.10 ^a^ 7.75 ^‡,c^	95 ± 5.7 94 ^‡c^	6.43 ^j^
betahis	5.49 ± 0.13	75 ± 2.0	5.84 ^‡,d^	86 ^‡,d^	
histapro	6.39 ± 0.03	33 ± 2.0	5.86 ± 0.07 ^a^ 6.95 ^‡,b^	31 ± 2.8 62 ^‡,b^	6.47 ^h^
Namh	5.56 ± 0.08	99 ± 2.0			
4mhis	4.46 ± 0.16	44 ± 2.4	4.80 ^‡,e^	90 ^‡,e^	
PI294	4.93 ± 0.03	29 ± 1.4	5.46 ^‡,f^	30 ^‡,f^	
suprahis	6.09 ± 0.13	49 ± 3.7	6.83 ^‡,b^	64 ^‡,b^	6.58 ^h^
dph	6.95 ± 0.04 (6.69) ± 0.17	−4 ± 0.1	(6.98) ± 0.07 ^a^ (7.81) ^‡,d^		7.40 ^k^
map	8.51 ± 0.04 (10.58) ± 0.11	−4 ± 0.2	(8.54) ^‡,g^		8.50 ^k^
mep	8.36 ± 0.11 (8.54) ± 0.19	−3 ± 0.2	(8.00) ± 0.17 ^a^ (8.25) ^‡,d^		8.39 ^k^ 8.7 ^l^
cyp	8.68 ± 0.24 (10.19) ± 0.10	−3 ± 0.5	(8.72) ^‡,d^		8.63 ^k^

Reference data are taken from (unless otherwise stated, E_max_ values refer to histamine = 100%): ^a^ functional [^35^S]GTPγS binding assays using *Sf*9 cells co-expressing either hH_1_R, Gα_q_, Gβ_1_ and Gγ_2_. ^‡,b–g^ functional [^32^P]GTPase activity assays using membrane preparations of *Sf*9 cells co-expressing hH_1_R and RGS4 (^b^ [12], ^c^ [40], ^d^ [47], ^e^ [48], ^f^ [49], ^g^ [50]). ^h,j^ [^3^H]mepyramine displacement assays using *Sf*9 cells co-expressing hH_1_R and RGS4 (^h^ [12], ^j^ [40]). ^k^ [^3^H]mepyramine displacement assays using HEK293T hH_1_R CRE-Luc cells expressing hH_1_R (^k^ [39]). ^l^ [^3^H]mepyramine displacement assays using whole cell homogenates of COS-7 cells expressing hH_1_R (^l^ [51]).

**Table 2:** Potencies (pEC_50_/p*K*_b_) and efficacies (E_max_) of ligands at the H_2_R explored in the mini-G protein recruitment assay. Data represent means ± SEM of at least three independent experiments (*n* ≥ 3), each performed in triplicate. Functional data obtained in steady-state GTPase assays and ligand binding affinities (p*K*_i_) determined in radioligand competition binding assays are included for comparison.

Compound	Mini G Protein Recruitment		GTPase		Competition Binding
	pEC_50_/(p*K*_b_)	E_max_ [%]	pEC_50_/(p*K*_b_)	E_max_ [%]	p*K*_i_
his	6.94 ± 0.05	100	6.00 ^a^	100 ^a^	6.27 ^d^
impro	7.48 ± 0.01	90 ± 1.5	6.80 ^a^	82 ^a^	6.3 ^e^
amt	7.57 ± 0.08	105 ± 2.8	6.72 ^a^	85 ^a^	6.61 ^d^
dim	6.47 ± 0.04	94 ± 2.6	6.04 ^a^	91 ^a^	4.6 ^e^
Namh	6.76 ± 0.09	93 ± 1.7			
4mhis	6.37 ± 0.05	93 ± 2.2	5.54 ^b^	101 ^b^	5.1 ^f^
PI294	6.92 ± 0.13	95 ± 1.1	6.43 ^c^	83 ^c^	
cim	6.02 ± 0.04 (6.28) ± 0.02	−8 ± 0.8	(5.77) ^a^	−8 ^a^	6.2 ^e^
fam	7.29 ± 0.10 (8.14) ± 0.09	−9 ± 0.7	(7.32) ^a^	−1 ^a^	7.8 ^e^ 6.87 ^d^
ran	7.02 ± 0.11 (6.99) ± 0.01	−8 ± 0.7	(6.08) ^a^	−9 ^a^	7.1 ^e^ 5.76 ^d^

Reference data are taken from (unless otherwise stated, E_max_ values refer to histamine = 100%): ^a–c^ functional [^32^P]GTPase activity assays using membrane preparations of *Sf*9 cells expressing a hH_2_R-Gα_s_ fusion protein (^a^ [11], ^b^ [48], ^c^ [49]). ^d^ [^3^H]UR-DE257 displacement assays using membrane preparations of *Sf*9 cells expressing a hH_2_R-Gα_s_ fusion protein (^d^ [52]). ^e,f^ [^125^I]iodoaminopotentidine displacement assays using membrane preparations of CHO cells expressing the hH_2_R (^e^ [53], ^f^ [54]).

**Table 3:** Potencies (pEC_50_/p*K*_b_) and efficacies (E_max_) of ligands at the H_3_R explored in the mini-Gprotein recruitment assay. Data represent mean values ± SEM of at least three independent experiments (*n* ≥ 3), each performed in triplicate. Statistical differences (*) of E_max_ > 100% was tested using a one-sample t-test (*n* = 5; α = 0.05). Functional data obtained in [^35^S]GTPγS and steady-state GTPase assays and ligand binding affinities (p*K*_i_, p*K*_d_) determined in radioligand competition/ saturation binding assays are included for comparison.

Compound	Mini G Protein Recruitment		GTPγS/GTPase ^‡^		Competition Binding
	pEC_50_/(p*K*_b_)	E_max_ [%]	pEC_50_/(p*K*_b_)	E_max_ [%]	p*K*_i_/(p*K*_d_)
his	6.47 ± 0.04	100	7.3 ^a^	89 ^a^	7.96 ^f^
imet	8.30 ± 0.17	67 ± 0.7	8.6 ^a^	80 ^a^	8.8 ^g^
immep	8.77 ± 0.05	63 ± 1.3	8.8 ^a^	77 ^a^	9.3 ^g^
VUF8430	5.21 ± 0.12	43 ± 1.6			6.0 ^h^
Namh	7.20 ± 0.03	111 ± 1.6 *	7.9 ^a^	100 ^a^	8.4 ^g^
4mhis	4.53 ± 0.08	19 ± 1.5			
PI294	8.40 ± 0.06	11 ± 1.1	8.80 ^‡,b^	39 ^‡,b^	(8.96) ^j^
thio	7.41 ± 0.04 (7.21) ± 0.07	−3 ± 0.4	6.9 ^a^	−52 ^a^	7.42 ^f^
clob	9.05 ± 0.10 (9.28) ± 0.12	−3 ± 0.2	9.14 ^‡,c^ (9.28) ^d^	−137 ^‡,c^	9.34 ^f^
JNJ	(5.44) ± 0.01				5.29 ^k^
pito	(8.41) ± 0.05		(9.80) ^e^		8.57 ^l^

Reference data are taken from (unless otherwise stated, E_max_ values refer to histamine = 100%): ^a^ functional [^35^S]GTPγS binding assays using membrane preparations of HEK293 cell expressing the hH_3_R (data normalized to (R)-α-methylhistamine (α = 100%) and ABT−239 (α = −100%) (^a^ [10]). ^‡,b,c^ functional [^32^P]GTPase activity assays using membrane preparations of *Sf*9 cells co-expressing hH_3_R, Gα_i2_ and Gβ_1_γ_2_ (^b^ [49], ^c^ [8]). ^d,e^ functional [^35^S]GTPγS binding assays using membrane preparations of CHO cells expressing the hH_3_R (^d^ [55], ^e^ [56]). ^f^ [^3^H]UR-PI294 displacement assays using membrane preparations of *Sf*9 cells co-expressing hH_3_R, Gα_i2_ and Gβ_1_γ_2_ (^f^ [57]). ^g,h,k^ [^3^H]*N*^α^-methylhistamine displacement assays using whole cell homogenates of SK-N-MC cells expressing the hH_3_R (^g^ [54], ^h^ [55] ^k^ [58]). ^j^ [^3^H]UR-PI294 saturation binding assay using membrane preparations of *Sf*9 cells co-expressing hH_3_R, Gα_i2_ and Gβ_1_γ_2_ (^j^ [57]). ^l^ [^125^I]iodoproxyfan displacement assay using whole cell homogenates of CHO cells expressing the hH_3_R (^l^ [56]).

**Table 4:** Potencies (pEC_50_/p*K*_b_) and efficacies (E_max_) of ligands at the H_4_R explored in the mini-G protein recruitment assay. Data represent mean values ± SEM of at least three independent experiments (*n* ≥ 3) each performed in triplicate. Functional data obtained in proximal [^35^S]GTPγS and steady-state GTPase and ligand binding affinities (p*K*_i_, p*K*_d_) determined in radioligand competition/saturation binding assays are included for comparison.

Compound	Mini G Protein Recruitment		GTPγS/GTPase ^‡^		Competition Binding
	pEC_50_/(p*K*_b_)	E_max_ [%]	pEC_50_/(p*K*_b_)	E_max_ [%]	p*K*_i_/(p*K*_d_)
his	6.40 ± 0.04	100	7.60 ^‡,a^	100 ^‡,a^	7.8 ^f^
imet	6.94 ± 0.04	47 ± 0.1	8.17 ^‡,b^	69 ^‡,b^	8.2 ^f^
immep	6.73 ± 0.05	66 ± 2.8	7.35 ^‡,b^	68 ^‡,b^	7.7 ^f^
VUF8430	6.47 ± 0.03	60 ± 0.2	7.42 ^c^	84 ^c^	7.5 ^f^
Namh	5.68 ± 0.06	82 ± 1.1			6.5 ^f^
4mhis	6.48 ± 0.06	78 ± 0.5	7.15 ^‡,d^	90 ^‡,d^	7.30 ^f^
PI294	7.71 ± 0.04	85 ± 0.6	8.35 ^c^	102 ^c^	(8.29) ^g^
clob	7.28 ± 0.06	48 ± 2.0	7.65 ^c^	45 ^c^	7.75 ^h^
thio	6.68 ± 0.04 (6.90) ± 0.01	−8 ± 1.9	6.58 ^c^ (6.83) ^c^	−139 ^c^	6.9 ^e^
JNJ	(7.25) ± 0.25	0 to 2.8	7.10 ^c^ (7.60) ^c^	−39 ^c^	7.52 ^h^
A943931	(8.43) ± 0.22	−4 to 2.8	7.3 ^e^	−180 ^e^	8.33 ^j^

Reference data are taken from (unless otherwise stated, E_max_ values refer to histamine = 100%): ^‡,a,b,d^ Steady-state GTPase activity assays using membrane preparations of *Sf*9 cells co-expressing hH_4_R, Gα_i2_ and Gβ_1_γ_2_ (data normalized to histamine = 100% and thioperamide = −100%^b^) (^a^ [59], ^b^ [60], ^d^ [48]) ^c,e^ [^35^S]GTPγS binding assays using membrane preparations of *Sf*9 cells co-expressing hH_4_R, Gα_i2_ and Gβ_1_γ_2_ (^c^ [9], ^e^ [61]). ^f^ [^3^H]histamine displacement assays using whole cell homogenates of SK-N-MC cells expressing the hH_4_R (^f^ [54]). ^g,h^ [^3^H]UR-PI294 saturation binding ^f^ and displacement ^g^ assays using membrane preparations of *Sf*9 cells co-expressing hH_4_R, Gα_i2_ and Gβ_1_γ_2_ (^g,h^ [57]). ^j^ [^3^H]histamine displacement assays using whole cell homogenates of HEK293 cells expressing the hH_4_R (^j^ [62]).

### 2.4. Influence of Mini-G Protein Co-Expression on Potencies and Dynamic Ranges

**Figure 3:**
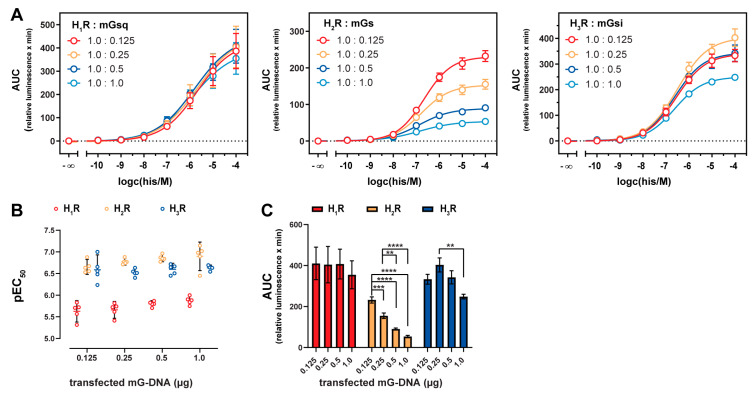
Concentration response curves and functional parameters obtained in the mini-G protein recruitment assay with different mini-G protein expression levels. (**A**) Concentration response curves, (**B**) pEC_50_ values and (**C**) AUCs of histamine obtained in the mini-G recruitment assay using HEK293T cells transiently transfected with indicated DNA amounts (in µg) of the H_1–3_R-NlucC and NlucN-mGsq/mGs/mGsi constructs 72 h prior to the experiments. Presented data are from five independent experiments (*n* = 5), each performed in triplicate. Whiskers (**B**) represent 95% confidential intervals. Significance levels (**C**) were calculated using one-way ANOVA followed by Tukey’s multiple comparison test calculated as ** *p* < 0.01, *** *p* < 0.005, **** *p* < 0.0001.

Mini-G proteins functionally mimic active Gα subunits and thus a mutual cooperativity between mini-G protein and agonist binding to GPCRs has been proposed [23]. We probed histamine at the H_1–3_ receptors co-expressed with increasing mini-G protein levels, but not at the H_4_R due to its weak transient expression. HEK293T cells were transiently transfected with constant receptor DNA amounts (1 µg) and increasing mini-G DNA amounts (0.125, 0.25, 0.5 and 1.0 µg) and were tested in the mini-G protein recruitment assay with histamine. In all three setups, the transfection of increasing mini-G gene doses were correlated with mini-G protein expression levels, which was demonstrated by a Western blot analysis (Supplementary Figure S5). In the mini-G protein recruitment assay, pEC_50_ values of histamine were not significantly shifted (α = 0.05) by increasing mini-G expression levels at the three receptor subtypes (Figure 3A,B) in contrast to suggestions of Wan et al. (2018) [23]. However, the signal amplitudes were affected differently for the three receptor/mini-G pairs. In the case of the H_1_R, the signal span was not altered by different mGsq expression levels (Figure 3A,C). On the contrary, the mGsi expression level determined by the highest gene dose of 1 µg significantly decreased the dynamic range at the H_3_R and, even more striking, all applied mGs gene doses in rising order led to significantly lowered dynamic ranges at the H_2_R (α = 0.05; Figure 3A,C). Similar to the collision coupling model of GPCR–G protein interaction [19,63], a possible explanation for the decreased signal amplitudes is that the basal activity of the histamine receptors increase due to higher mini-G expression levels and, thus, a more likely collision of constitutively active receptors and the respective mini-G protein. However, one has to be careful judging the extent of the signal span reduction observed for the H_1_R/mGsq, H_2_R/mGs and H_3_R/mGsi pairs, as e.g., same gene doses of mGsq led to considerably lower expression levels compared to mGs in the Western blot analysis (Supplementary Figure S5) [23].

### 2.5. Stabilization of the Active H_2_R Conformation by the Minimal Gα_s_ Protein

**Figure 4:**
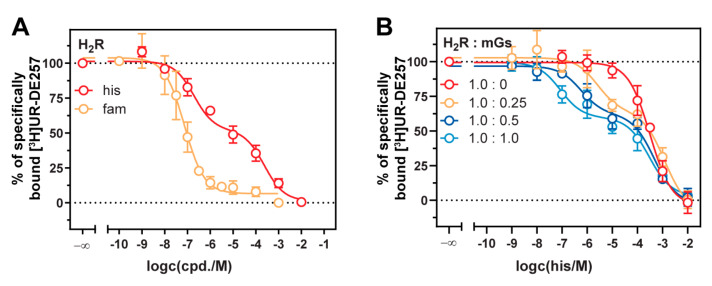
Comparison of radioligand displacement curves at cells co-expressing the H_2_R and mGs. (**A**) [^3^H]UR-DE257 (50 nM) was displaced by either histamine or famotidine. Presented data are means ± SEM of three independent experiments (*n* = 3), each performed in triplicate using HEK293T cells stably co-expressing the H_2_R-NlucC and NlucN-mGs constructs. (**B**) Displacement of [^3^H]UR-DE257 (50 nM) by histamine using HEK293T cells transiently transfected with indicated DNA amounts (in µg) of the H_2_R-NlucC and NlucN-mGs constructs 72 h prior to the experiments. Presented data are means ± SEM of three independent experiments (*n* = 3), each performed in duplicate.

As the signal amplitude at the H_2_R could be correlated to the mGs expression (Figure 3), we further explored binding properties of the endogenous agonist histamine and the antagonist famotidine by displacement of [^3^H]UR-DE257 at HEK293T cells stably expressing the NlucN-mGs and H_2_R-NlucC fusion proteins (Figure 4A, Supplementary Table S2). Whereas the radioligand displacement by famotidine followed a monophasic curve supporting a one-site binding model (p*K*_i_ = 7.68 ± 0.01), notably a two-sites binding model was preferred for the agonist histamine (p*K*_i,low_ = 3.87 ± 0.13; p*K*_i,high_ = 6.94 ± 0.14). Thus, we assumed there was a high affinity binding site at the H_2_R as previously described for the ternary H_2_R-G protein complex [53]. To correlate the observation to the amount of co-expressed mGs, we probed the binding of histamine at the H_2_R by transient transfections of increasing mGs gene doses (from 0 µg to 1 µg of mGs DNA) and a constant gene dose of H_2_R (1 µg; Figure 4B, Supplementary Table S2). The expression of the H_2_R alone (0 µg of mGs DNA) led to a rightward shifted, but monophasic concentration response curve of histamine. In contrast, by increasing mGs gene doses, we recorded an extended formation of the high affinity binding site (Figure 4B, Supplementary Table S2). Therefore, we deduce that mGs stabilizes the active conformation of the H_2_R in a concentration-dependent manner. Although endogenously expressed G proteins are also intended to stabilize active receptor conformations, we did not detect a high affinity binding site using HEK293T cells that were transiently transfected with the H_2_R alone. On the one hand, this could be traced back to the lower native expression levels of G proteins compared to the overexpressed mGs. On the other hand, mGs constitutes the active GTPase domain of Gα_s_ and therefore is immediately accessible for binding to the H_2_R in active state, whereas endogenous G proteins presumably exist in diverse conformations [53].

## 3. Discussion

Our study focused on the development of a novel live cell assay that reports on functional properties of histamine receptor ligands at an early stage of signal transduction. We achieved this by applying the split-NanoLuc to the four histamine receptors and minimal (chimeric) G proteins. We observed excellent signal amplitudes at all four receptor subtypes, which was of particular importance for the weakly expressed recombinant H_4_R. Moreover, we are the first to provide time-resolved courses of agonist-mediated functional responses using mini-G sensors with split-NanoLuc complementation for an entire receptor family, the subtypes of which couple to three different types of mini-G proteins (mGs, mGsi and mGsq). As the presented biosensor is becoming available for an increasing number of GPCRs [23,24,25,26,27], it will be appealing to extend the application in prospective studies to analyse time-resolved differences of distinct GPCRs coupling to the same minimal G protein. The methodology could also be used to investigate one GPCR coupling to different minimal G proteins (coupling specificity), and to supplement ligand binding studies with kinetic input, for example association and dissociation rate constants (*k*_on/off_) and residence time [32,33]. This might contribute to an even better pharmacological understanding of receptor regulation, as well as signal formation and transduction [64,65].

By investigating a large set of standard ligands, we demonstrated the usefulness of the mini-G sensor to reliably characterize agonists and antagonists. In our system, all four histamine receptor subtypes were constitutively active, although to a lesser extent than reported in other recombinant systems with the H_4_R [46,60]. The occurrence of such constitutively active receptors depends on the expression levels and the stoichiometry of the GPCRs and the G proteins according to the extended ternary complex (ETC) model of GPCR function [66]. Thus, the applied test system limits the detectability of the constitutive activity and the extent of the inverse efficacy of a ligand [67]. In future routine characterization of histamine receptor ligands, it would be convenient to introduce a reference ligand that produces inverse deflection of bioluminescence in the mini-G protein recruitment assay, such as diphenhydramine (H_1_R), famotidine (H_2_R) and thioperamide (H_3_R, H_4_R).

In the literature, two models of the GPCR-G protein interaction are discussed: a collision coupling, and a pre-coupled model [19,20,63]. In the case of the H_2_R, the lower the gene dose of the mGs, the higher the dynamic range. Further, we observed a high affinity binding site for the agonist histamine subject to the mGs expression level in radioligand competition binding experiments. Both results agreed with the collision coupling model of GPCR-G protein interaction, which supports an increased constitutive activity of GPCRs highly expressed in recombinant systems [19,63]. Therefore, it was not surprising that we did not detect such correlation for the H_1_R and H_3_R. Both receptors were expressed to a lesser extent compared to the H_2_R (Supplementary Table S1) and also the expression level of mGsq was considerably lower compared to mGs (Supplementary Figure S5).

Concisely, this study describes the establishment and usefulness of a mini-G sensor for prospective drug discovery at the histamine receptors. Due to the homogenous nature and the non-radioactive readout with an, per definition, excellent dynamic range (Z’ factor), this assay will be automatable, and should be compatible with HTS.

## 4. Materials and Methods

### 4.1. Materials

Dulbecco’s modified Eagle’s medium (DMEM) was purchased from Sigma-Aldrich (Taufkirchen, Germany) and Leibovitz’ L-15 medium (L-15) from Fisher Scientific (Nidderau, Germany). FBS, trypsin/EDTA and geneticin (G418) were from Merck Biochrom (Darmstadt, Germany), whereas puromycin was from InvivoGen (Toulouse, France) and furimazine from Promega (Mannheim, Germany). The pcDNA3.1 vector was from Thermo Scientific (Nidderau, Germany) and the pIRESpuro3 vector was a kind gift from Prof. Dr. Gunter Meister (University of Regensburg). Histamine dihydrochloride (his) was purchased from Tokyo Chemical Industry (Eschborn, Germany), whereas 4-methylhistamine dihydrochloride (4mhis), mepyramine maleate (mep), imetit dihydrobromide (imet), immepip dihydrobromide (immep), thioperamide maleate (thio), clobenpropit dihydrobromide (clob) and A943931 dihydrochloride (A943931) were from Tocris Bioscience (Bristol, United Kingdom). *N*^α^-methylhistamine dihydrochloride (Namh), betahistine dihydrochloride (betahis), diphenhydramine hydrochloride (dph), maprotiline hydrochloride (map), cyproheptadine hydrochloride sesquihydrate (cyp), amthamine dihydrobromide (amt), dimaprit dihydrochloride (dim), cimetidine (cim), famotidine (fam) and ranitidine hydrochloride (ran) were purchased from Sigma. Histaprodifen [68] (histapro), suprahistaprodifen [68] (suprahis), UR-KUM530 [12] (KUM530), impromidine [69] (impro), UR-PI294 [49] (PI294), VUF8430 [55] (VUF8430) and JNJ7777120 [70] (JNJ) were synthesized in-house according to published procedures. Pitolisant hydrochloride (pito) was kindly provided by Prof. Dr. Katarzyna Kiec-Kononowicz (Jagiellonian University, Krakow). All ligands were dissolved, according to their physicochemical properties. Preferebly, the ligands were dissolved in Millipore water, except for histaprodifen (histapro), suprahistaprodifen (suprahis), maprotiline (map), cimetidine (cim) and famotidine (fam). In these cases, DMSO (Merck) was (proportionally) used as solvent (DMSO/H_2_O: histapro, suprahis: 50/50; map: 30/70; cim, fam: 100% DMSO).

### 4.2. Molecular Cloning

The human codon-optimized cDNA fragments encoding the mini-G proteins mGs, mGsi and mGsq (corresponding to mini-Gs393, mini-Gs/i43 and mini-Gs/q71 published by Nehmé [22], Supplementary Figure S1), were synthesized by Eurofins Genomics (Eurofins Genomics LLC, Ebersberg, Germany). Plasmids containing the split-NanoLuc fragments (NlucN, 159 amino acids; NlucC, 11 amino acids) were from Promega and cDNAs encoding the histamine receptors were purchased from the Missouri cDNA research center (Rolla, MO, USA). All cDNAs were amplified by PCR and subcloned into vector backbones by standard molecular cloning techniques. For this purpose, a set of pIRESpuro3 vectors was generated encoding the respective mini-G protein, which was N-terminally fused to the large split-luciferase fragment (NlucN) separated by a flexible glycine-serine-linker (encoding -GSSGGGGSGGGGSS-). The sequence encoding the H_1_R-NlucC described by Littmann et al. (2019) was subcloned into pcDNA3.1 using the restriction enzymes *Hin*dIII and *Sa*cII, and the receptor sequence was then replaced by either the H_2_R, H_3_R or H_4_R gene using *Hin*dIII and *Xb*aI [71]. The optimal arrangement of a split-luciferase system to study the interaction of GPCRs and intracellular proteins of interest (GPCR-NlucC and NlucN-protein) was reported previously [23,71]. Plasmid DNA was quantified by UV-Vis absorbance using a NanoDrop spectrophotometer (ThermoFisher, Braunschweig, Germany). All sequences were verified by sequencing performed by Eurofins Genomics.

### 4.3. Cell Culture

HEK293T cells were a kind gift from Prof. Dr. Wulf Schneider (Institute for Medical Microbiology and Hygiene, Regensburg, Germany) and cultured in DMEM supplemented with 10% FBS at 37 °C in a water-saturated atmosphere containing 5% CO_2_. Cells were periodically inspected for mycoplasma contamination by means of the Venor GeM Mycoplasma Detection Kit (Minerva Biolabs, Berlin, Germany) and proven negative.

### 4.4. Generation of Stable Transfectants

In order to generate stable cell lines, wildtype HEK293T cells were stepwise transfected with a pIRESpuro3 vector encoding either the NlucN-mGs, -mGsi or -mGsq protein, and with the respective pcDNA3.1 plasmid encoding the histamine H_1–4_ receptor-NlucC fusion protein according to the XtremeGene HP transfection protocol (Merck). The cells were then cultured in DMEM supplemented with 10% FBS, 1 µg/mL puromycin and 600 µg/mL G418 for sustained selection pressure.

### 4.5. Generation of Transient Transfectants

Adjusted to a cell density of 0.3 × 10^6^ cells/mL, HEK293T cells were seeded into a 6-well cell culture plate (Sarstedt, Nürnbrecht, Germany) and allowed to attach overnight. The next day, the cells were transfected using linear polyethyleneimine (PEI, 1 mg/mL in PBS; 1:5 ratio (2 µg DNA: 10 µL PEI)) and incubated for another 48 h to allow for adequate protein expression. For mini-G protein recruitment assays and radioligand competition binding experiments, we applied a constant amount of 2 µg of total DNA per 6-well (total volume of 2 mL) comprising 1 µg of pcDNA3.1 H_1/2/3_R-NlucC and increasing amounts of the pIRESpuro3 NlucN-m/Gsq/mGs/mGsi DNA (0.125, 0.25, 0.5, or 1.0 µg). To ensure a uniform transfection efficiency, the empty pIRESpuro3 vector was co-transfected as mock DNA (0.875, 0.75, 0.5 µg or none). For Western blot analysis of the mini-G protein expression, the cells were transfected with a total amount of 2 µg DNA comprising 0.125, 0.25, 0.5 or 1.0 µg of the pIRESpuro3 NlucN-m/Gsq/mGs/mGsi and 1.875, 1.750, 1.5 and 1.0 µg, respectively, of the empty pIRESpuro3 vector as mock DNA.

### 4.6. Western Blot Analysis

Cells were lysed using a RIPA lysis buffer (50 mM Tris, 0.1% sodium dodecyl sulfate, 0.5% sodium deoxycholate, 1% Triton X-100, 150 mM NaCl) supplemented with SIGMAFAST protease inhibitor cocktail tablets according to the manufacturer’s protocol (Sigma-Aldrich). Lysates (15 µg protein) and 10 µL of the Precision Plus Protein^TM^ Dual Color Standard (Bio-Rad, Feldkirchen, Germany) were loaded to an 8–16% Novex Tris-glycine polyacrylamide gel (Thermo Scientific) and SDS-page was performed at 225 V for 1 h. Thereafter, the proteins were blotted on a nitrocellulose membrane (0.2 A, 1 h). By incubation with 5% skim milk powder in phosphate-buffered saline supplemented with 0.05% Tween 20 (PBS-T) for 1 h at RT, nonspecific binding sites of the membrane were blocked. After three washing steps with PBS-T, blots were incubated overnight at 4 °C with the primary antibodies α-Nluc (1:5000; in PBS-T; polyclonal, produced in rabbit, kindly provided by Promega) and α-vinculin (1:500; in PBS-T; monoclonal; MAB6896, produced in mouse, R&D Systems Inc., MN, USA). After additional three washing steps on the next day, the membranes were incubated with the HRP-conjugated secondary antibodies (raised against IgG, respectively) α-rabbit (1:10,000 in PBS-T; sc-2313, produced in donkey, Santa Cruz, TX, USA) and α-mouse (1:100,000 in PBS-T; A0168, produced in goat; Sigma-Aldrich) for 3 h at RT. The blots were washed three times with PBS-T and developed using the Clarity Western ECL substrate (Bio-Rad, Feldkirchen, Germany). Subsequently, the colorimetric and luminescent images of the stained blots were captured using a ChemiDoc MP imager (Bio-Rad).

### 4.7. Mini-G Protein Recruitment Assay

The day before the experiment, cells were detached by trypsinization (0.05% trypsin, 0.02% EDTA in PBS) and centrifuged (700 g, 5 min). Subsequently, the cells were resuspended in L-15 supplemented with 10 mM HEPES (Serva, Heidelberg, Germany) and 5% FBS. Thereafter, 100.000 cells per well were seeded onto a white flat-bottom 96-well microtiter plate (Cat. No. 781965, Brand GmbH + CoKG, Wertheim, Germany) and incubated at 37 °C in a water-saturated atmosphere without additional CO_2_ overnight. Shortly before the experiment, the substrate furimazine was diluted in L-15 and 10 µL were added to the cells (final dilution 1:1000). Then, the plate was transferred to a pre-heated (37 °C) EnSpire plate reader (Perkin Elmer Inc., Rodgau, Germany). After recording the basal luminescence for 15 min, 10 µL of the agonist serial dilutions were added to the cells (final volume: 100 µL) and luminescence traces were recorded for 45 min (agonist mode). When investigating antagonists, the antagonist dilutions were added before the reference agonist histamine (EC_80_ concentration; H_1_R: 10 µM, H_2–4_R: 1 µM) and the cells were incubated for 15 min (antagonist mode). Luminescence was captured with an integration time of 0.1 s per well. Data were analyzed using GraphPad Prism8 software (San Diego, CA, USA). The relative luminescence units (RLU) were corrected for (slight) inter-well variation caused by differences in cell density and substrate concentration, as well as for baseline drift, by dividing all data by the mean luminescence intensity of the respective L-15 control. AUCs of the luminescence traces for each concentration were calculated and normalized to the maximum response of 100 µM histamine (100% control) and L-15 (0% control). The logarithmic ligand concentrations were fitted against the normalized intensities with variable slope (log(c) vs. response–variable slope (four parameters)). The fit yielded pEC_50_ and E_max_ values in the case of agonists, and pIC_50_ values in the case of antagonists, which were used to calculate p*K*_b_ values according to the Cheng-Prusoff-equation [72]. In order to assess Z’ factors, the baseline-corrected relative luminescence units (RLU) of 100 µM histamine and L-15 were inter-well corrected and AUCs were used for the calculation of means and standard deviations [34].

Significant differences in the efficacies obtained in the mini-G protein recruitment assay were assessed using a one-sample t-test (*n* = 5; α = 0.05). When investigating the influence of the mini-G protein expression level, significant differences between AUCs and pEC_50_ values were calculated using one-way ANOVA followed by Tukey’s multiple comparison test (*n* = 5, α = 0.05).

### 4.8. Radioligand Binding Experiments

Radioligand saturation binding experiments were performed using intact HEK293T cells co-expressing either NlucN-mGsq/H_1_R-NlucC, NlucN-mGs/H_2_R-NlucC, NlucN-mGsi/H_3_R-NlucC or NlucN-mGsi/H_4_R-NlucC. The following radioligands were used to verify the receptor expressions: [^3^H]mepyramine (a_s_ = 20 Ci/mM, Hartmann Analytics GmbH, Braunschweig, Germany) for the H_1_R, [^3^H]UR-DE257 [52] (a_s_ = 32.9 Ci/mmol) for the H_2_R and [^3^H]UR-PI294 [57] (a_s_ = 93.3 Ci/mmol) for the H_3_R and H_4_R. The specific binding of each radioligand was determined by subtracting the non-specific binding from the corresponding total binding. The cells were incubated with various concentrations of the radioligands in the absence (L-15) (total binding) or presence of a competitor at a final concentration of 10 µM (nonspecific binding). As competitors, we applied diphenhydramine for the H_1_R, famotidine for the H_2_R, thioperamide for the H_3_R or histamine for the H_4_R. Radioligand competition binding experiments were performed using intact HEK293T cells expressing the NlucN-mGs and H_2_R-NlucC fusion proteins. The cells were incubated with 50 nM [^3^H]UR-DE257 and with the ligands in serial dilution and with L-15 (negative control). The non-specific binding of the radioligand was determined in the presence of famotidine at a final concentration of 10 µM and subtracted from all values.

For both, radioligand saturation and competition binding experiments, all (radio)ligand dilutions were prepared 10-fold concentrated in L-15 and 10 µL/well were transferred to a round bottom polypropylene 96-well microtiter plate (Greiner Bio-One, Frickenhausen, Germany). The cells were detached by trypsinization (0.05% trypsin + 0.02% EDTA), harvested by centrifugation (700 g, 5 min) and resuspended in L-15. The cells were adjust-ed to a density of 1.0 × 10^6^ cells/mL and 80 µL of the cell suspension were added to each well (final assay volume of 100 µL). Then, the cells were incubated at room temperature under shaking for 60–120 min, and the cells were collected by filtration and washed with ice-cold PBS using a 96-well harvester (Brandel Inc., Unterföhring, Germany). The cell-associated radioactivity was measured by liquid scintillation counting, as previously described [73].

All data were analyzed using GraphPad Prism8 software. In the case of saturation binding experiments, all data were best fitted to a one-site saturation binding model (one site—total and nonspecific binding; one site—specific binding) yielding *K*_d_ values. For competition binding experiments, data of the agonist histamine were best fitted to a two-sites competition binding model (two sites—fit logIC50) yielding pIC_50,high_ and pIC_50,low_. Except, competition binding data of histamine using cells transiently transfected with the H_2_R alone and data of the antagonist famotidine obtained at cells stably co-expressing the H_2_R and mGs were fitted to the one-site three parameter logistic fit (one-site—fit logIC50) to determine pIC_50_ values. Obtained pIC_50_ values (pIC_50_, pIC_50,high_, pIC_50,low_) were then used to calculate p*K*_b_ values according to the Cheng-Prusoff-equation [72].

## Supplementary Materials

Supplementary materials can be found at https://www.mdpi.com/1422-0067/21/22/8440/s1.

## Abbreviations

H_1_R	Human histamine H_1_ receptor
H_2_R	Human histamine H_2_ receptor
H_3_R	Human histamine H_3_ receptor
H_4_R	Human histamine H_4_ receptor
HEPES	4-(2-Hydroxyethyl)piperazine-1-ethanesulfonic acid
L-15	Leibovitz’ L-15 medium
NanoLuc	NanoLuc luciferase
