# Supplementary material for: A Dynamic, Split-Luciferase-Based Mini-G Protein Sensor to Functionally Characterize Ligands at All Four Histamine Receptor Subtypes
Source: Int J Mol Sci. 2020 Nov 10;21(22):8440. doi: 10.3390/ijms21228440 (PMC7698210; doi:10.3390/ijms21228440)
Supplement: Supplementary file 1 [file ijms-21-08440-s001.pdf]

Supporting Material

A dynamic, split-luciferase-based mini-G protein sensor to functionally characterize ligands at all four histamine receptor subtypes.

Carina Höring*, Ulla, Seibel, Katharina Tropmann, Lukas Grätz, Denise Mönnich, Sebastian Pitzl, Günther Bernhardt, Steffen Pockes, Andrea Strasser*

Institute of Pharmacy, Faculty of Chemistry and Pharmacy, University of Regensburg, Regensburg, Germany

*Correspondence to: carina.hoering@ur.de and andrea.strasser@ur.de

Contents

Supplementary Figure S1	Sequences of utilized mini-G proteins.	p. 3
Supplementary Figure S2	Exemplary radioligand saturation binding curves with HEK293T cells stably co-expressing the histamine $H_{1\text{-}4}$ receptors in combination with either mGsq, mGs or mGsi.	p. 4
Supplementary Figure S3	Representative signals used for the determination of the Z′ factor.	p. 5
Supplementary Figure S4	Structures of investigated histamine receptor ligands.	p. 6
Supplementary Figure S5	Western blot analysis of HEK293T cell lysates expressing the NlucN-mG fusion proteins.	p. 7
Supplementary Table S1	pK_d values of radioligands determined in saturation binding experiments using HEK293T cells stably co-expressing either the H_1R-NlucC/ NlucN-mGsq, H_2R-NlucC/ NlucN-mGs, H_3R-NlucC/ NlucN-mGsi or H_4R-NlucC/ NlucN-mGsi.	p. 8
Supplementary Table S2	Binding affinities (pK_i, $pK_{i,low}$, $pK_{i,high}$)of ligands at the H_2R.	p. 9
Supplementary Method	[^{35}S]GTPγS binding assay at the H_1R (protocol).	p. 10
	References	p. 11

Supplementary Figure S1

```
>mGs (mini-Gs393)
MIEKQLQKDKQVYRATHRLLLLGADNSGKSTIVKQMRILHGGSGGSGGTSGIFETKFQVDKVNFHMFDVGGQRD
ERRKWIQCFNDVTAIIFVVDSSDYNRLQEALNDFKSIWNNRWLRTISVILFLNKQDLLAEKVLAGKSKIEDYFPEFAR
YTTPEDATPEPGEDPRVTRAKYFIRDEFLRISTASGDGRHYCYPHFTCAVDTENARRIFNDCRDIIQRMHLRQYELL
>mGsi (miniGs/i43)
MIEKQLQKDKQVYRATHRLLLLGADNSGKSTIVKQMRILHGGSGGSGGTSGIFETKFQVDKVNFHMFDVGGQRD
ERRKWIQCFNDVTAIIFVVDSSDYNRLQEALNDFKSIWNNRWLRTISVILFLNKQDLLAEKVLAGKSKIEDYFPEFAR
YTTPEDATPEPGEDPRVTRAKYFIRDEFLRISTASGDGRHYCYPHFTCAVDTENARRIFNDVTDIIIKMNLRDCGLF
>mGsq (miniGs/q71)
MIEKQLQKDKQVYRRTLRLLLLGADNSGKSTIVKQMRILHGGSGGSGGTSGIFETKFQVDKVNFHMFDVGGQRD
ERRKWIQCFNDVTAIIFVVDSSDYNRLQEALNDFKSIWNNRWLRTISVILFLNKQDLLAEKVLAGKSKIEDYFPEFAR
YTTPEDATPEPGEDPRVTRAKYFIRKEFVDISTASGDGRHICYPHFTCAVDTENARRIFNDCKDIILQMNLREYNLV
```

Sequences of utilized mini-G proteins.

Supplementary Figure S2

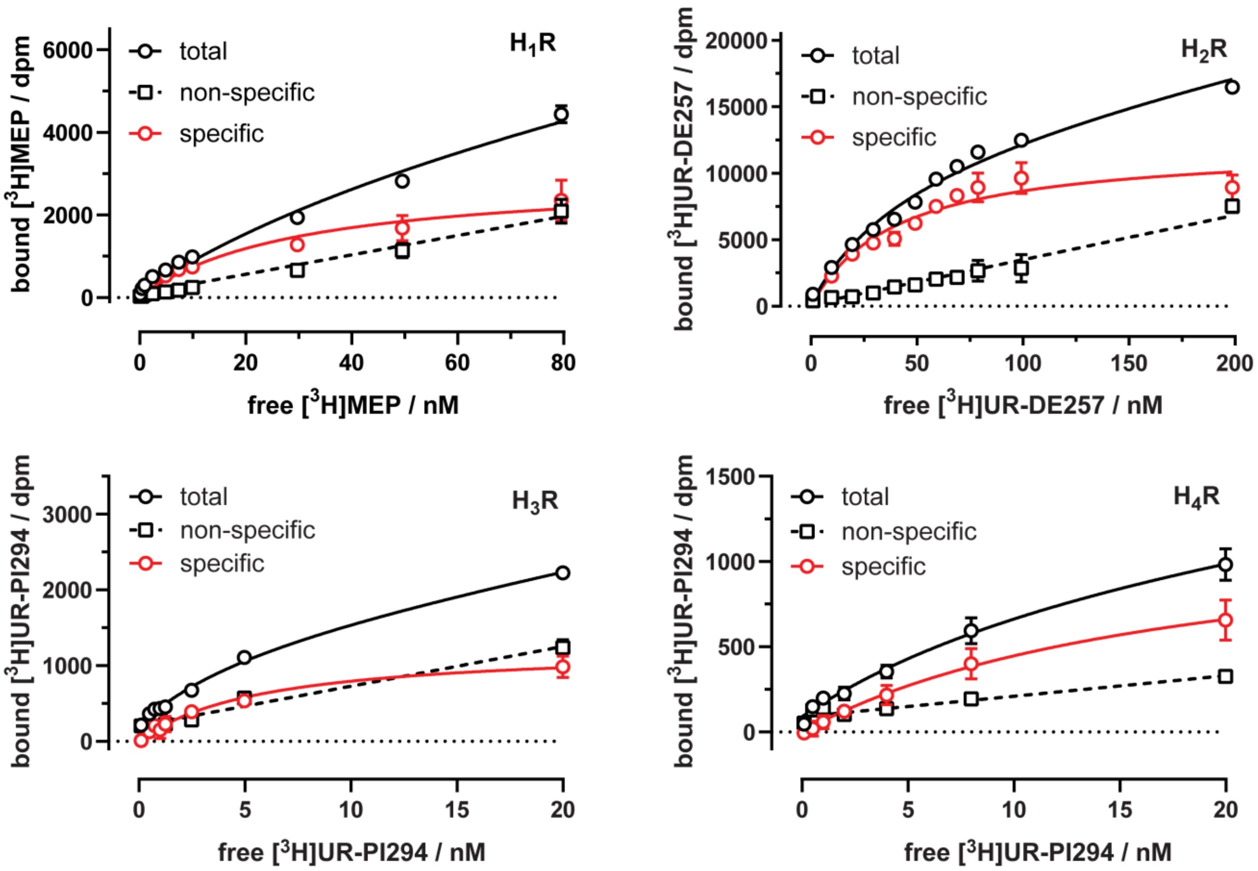

Exemplary radioligand saturation binding curves with HEK293T cells stably co-expressing the histamine H_{1-4} receptors in combination with either mGsq, mGs or mGsi. Representative data from saturation binding experiments of $[^3H]$mepyramine ($[^3H]$MEP) at the H_1R, $[^3H]$UR-DE257 at the H_2R and $[^3H]$UR-PI294 at the H_3R and H_4R. The nonspecific binding of each radioligand concentration was determined in the presence of either 10 µM diphenhydramine (H_1R), famotidine (H_2R), histamine (H_3R) or thioperamide (H_4R), respectively.

Supplementary Figure S3

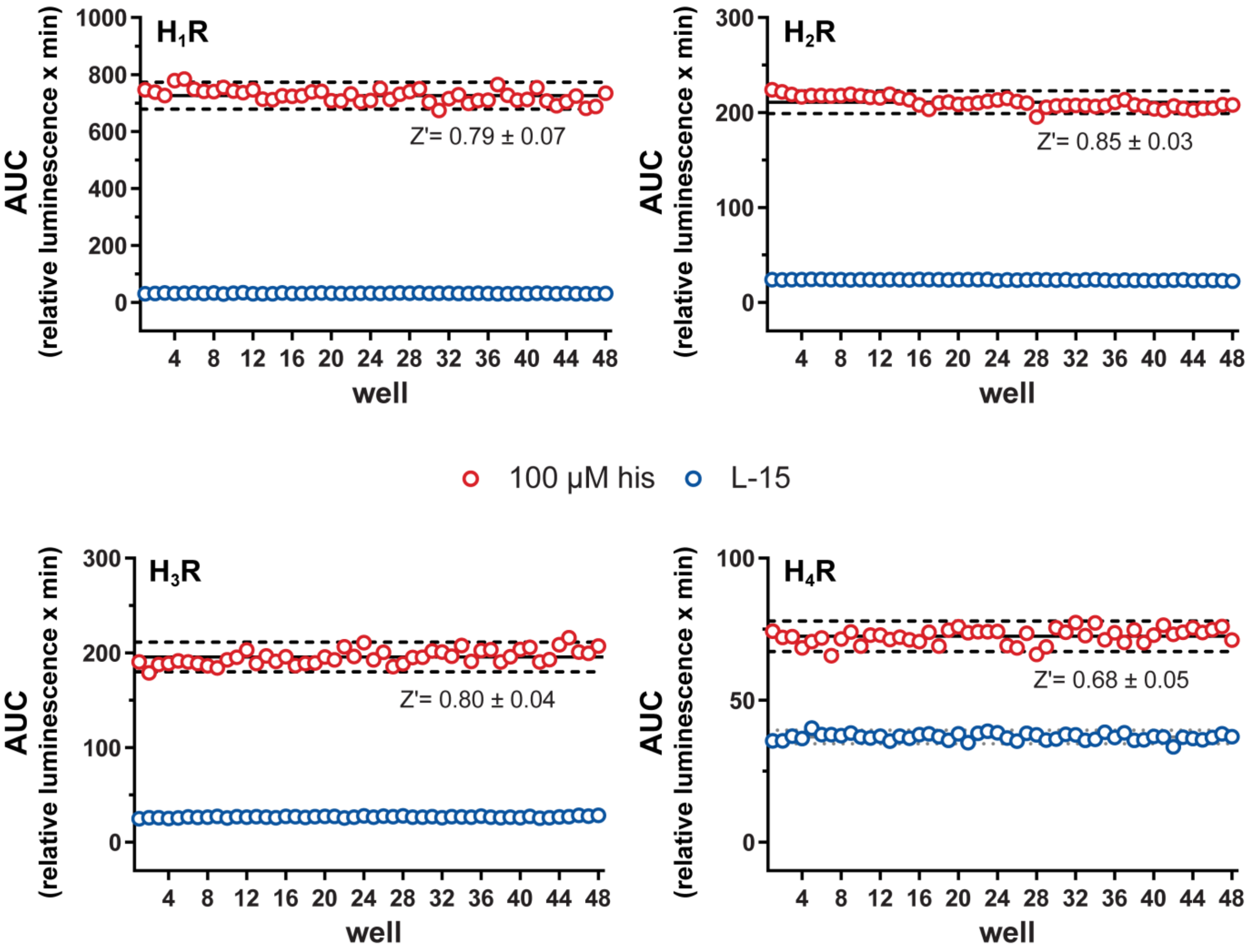

Representative signals used for the determination of the Z′ factor. Area under curves (AUC) of 100 µM histamine and Leibovitz′ L-15 as buffer control for each well are plotted. Presented data are from representative plates. Indicated Z′ are mean ± SEM of at least three experiments (*N* = 3).

Supplementary Figure S4

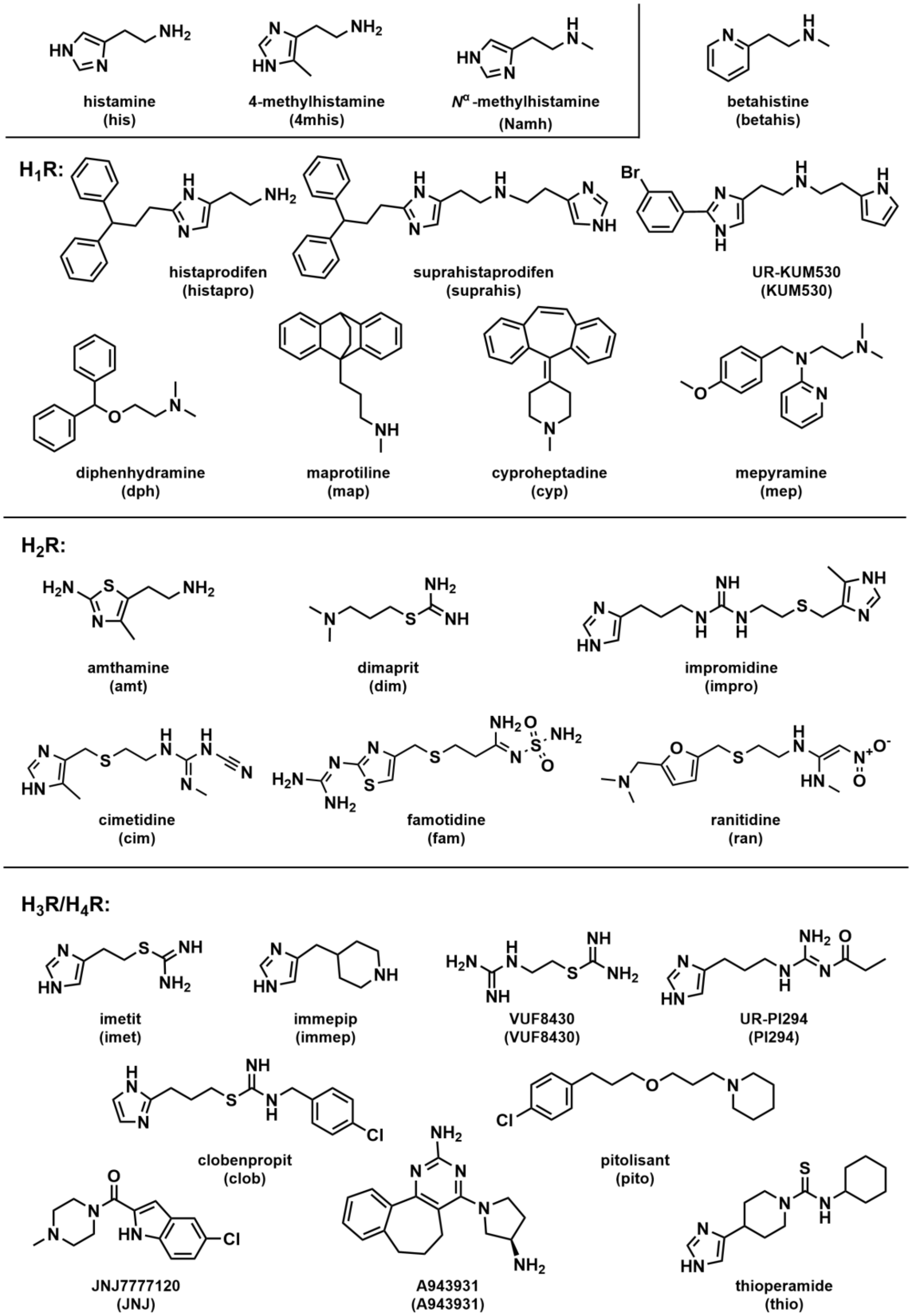

Structures of investigated histamine receptor ligands.

Supplementary Figure S5

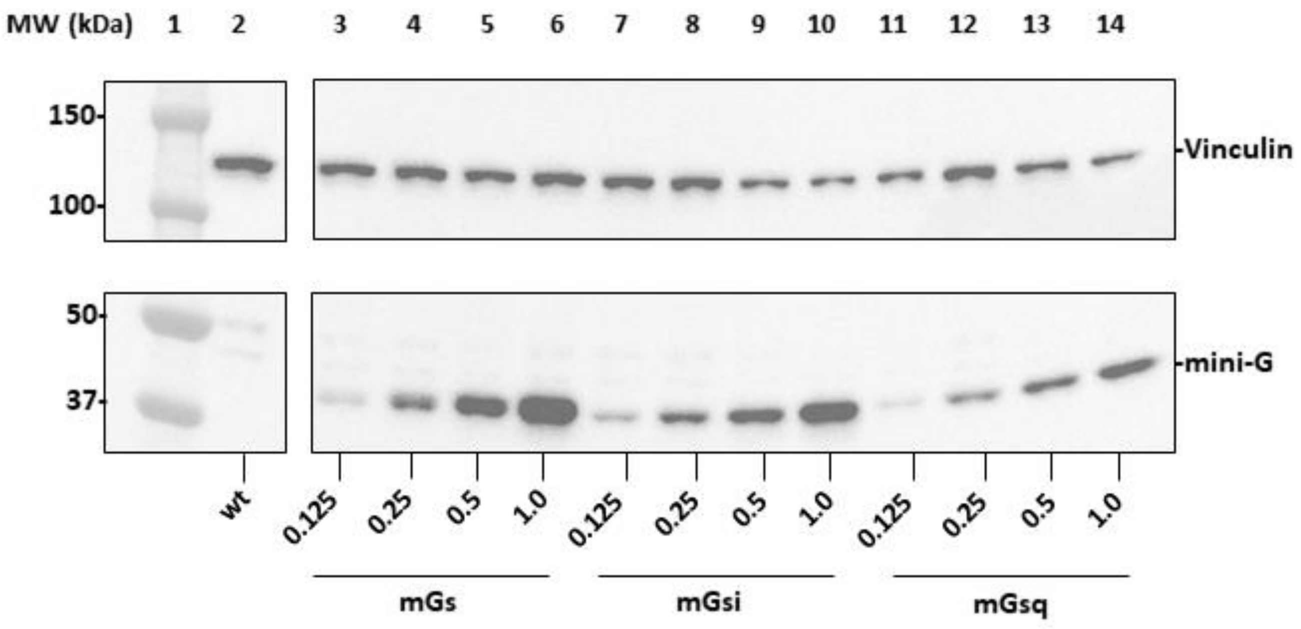

Western blot analysis of HEK293T cell lysates expressing the NlucN-mini-G fusion proteins. The cells were transiently transfected with indicated plasmid DNA (µg) encoding the NlucN-mGs,-mGsi, and -mGsq fusion proteins. For primary staining, α-Nluc (rat; 1:5,000 in PBS-T) and α-vinculin (mouse; 1:500 in PBS-T) antibodies were used and for secondary staining α-rabbit (HRP-conjugated; 1:10,000 in PBS-T) and α-mouse (HRP-conjugated; 1:100,000 in PBS-T) were used, both raised against IgG. A lysate of HEK293T wildtype (wt) cells was used as negative control. Shown is a superposition of the colorimetric and Chemi Hi resolution images captured with a ChemiDoc MP imager (Bio-Rad).

Supplementary Table S1

pK_d values of radioligands determined in saturation binding experiments using HEK293T cells stably co-expressing either the H_1R-NlucC/ NlucN-mGsq, H_2R-NlucC/ NlucN-mGs, H_3R-NlucC/ NlucN-mGsi or H_4R-NlucC/ NlucN-mGsi. Presented data are mean ± SEM of at least three experiments (*N* = 3) conducted in triplicate.

receptor	radioligand saturation binding				reference	
	radioligand	pK_d ± SEM	B_{max} ± SEM	sites / cell	pK_d	
H_1R	[^{3}H]mepyramine	7.59 ± 0.08	2224 ± 364	377111 ± 61750	8.35[a]	[1]
H_2R	[^{3}H]UR-DE257	7.35 ± 0.08	10070 ± 1664	1038127 ± 171508	7.26[b]	[2]
H_3R	[^{3}H]UR-PI294	8.37 ± 0.12	1130 ± 118	41103 ± 4300	8.96[c]	[3]
H_4R	[^{3}H]UR-PI294	7.85 ± 0.07	1568 ± 503	50848 ± 12349	8.29[c]	[3]

Reference pK_d values were obtained for the indicated radioligands in radioligand saturation binding experiments using membrane preparations of *Sf*9 cells (co-) expressing either the hH_1R and RGS4[a], hH_2R-$G\alpha_s$ fusion protein[b], or hH_3R, $G\alpha_{i2}$ and $G\beta_1\gamma_2$[c].

Supplementary Table S2

Binding affinities (pK_i, p$K_{i,low}$, p$K_{i,high}$) of ligands at the H_2R. 50 nM [^{3}H]UR-DE257 were displaced by the respective ligand. Utilized HEK293T cells either stably co-expressed the H_2R-NlucC and NlucN-mGs constructs or were transiently transfected with the indicated cDNA amounts (µg; H_2R + mGs) of the latter. Data represent means ± SEM of at least three independent experiments each performed in triplicate (stable transfectants) and duplicate (transient transfectants).

**cell line**	**radioligand competition binding**				**reference**
**(µg cDNA)**	**compound**	**p$K_{b,low}$ ± SEM**	**p$K_{b,high}$ ± SEM**	**pK_b ± SEM**	**pK_b**
stable	his	3.87 ± 0.13	6.94 ± 0.14	--	6.27[a] [2], 4.37[b] [4]
stable	fam	--	--	7.68 ± 0.01	7.8[c] [5]
transient (1.0 + 0.0)	hist	--	--	3.55 ± 0.09	
transient (1.0 + 0.25)	his	3.14 ± 0.17	5.98 ± 0.12	--	
transient (1.0 + 0.5)	his	3.63 ± 0.80	6.49 ± 0.08	--	
transient (1.0 + 1.0)	his	3.90 ± 0.18	7.37 ± 0.12	--	

Reference data is reported from experiments using membrane preparations of *Sf*9 cells expressing a hH_2R-$G\alpha_s$ fusion protein[a,b] or CHO cells expressing the hH_2R[c]. For [b] 145 mM NaCl was added to the assay buffer. Indicated pK_i values were assessed by displacement of [^{3}H]UR-DE257[a] or [^{125}I]iodoaminopotentidine[c].

Supplementary Method

[^{35}S]GTPγS binding assay at the H_1R (protocol).

Cloning and protein expression.

The pcDNA3.1 vector encoding the human H_1 receptor, the $G\alpha_q$, the $G\beta_1$ or the $G\gamma_2$ sequences were from the cDNA Resource Center (Rolla, MO, USA). For cloning of H_1R into the pFastBac1 vector [6], the receptor was amplified using the PCR protocol for Phusion® DNA polymerase New England Biolabs, Frankfurt a. M.). A *Bam*HI restriction site was added at the 5'-end, followed by HA and FLAG tag and a *Hin*dIII restriction site at the 3'-end. This construct was inserted into the linearized vector according to the NEBuilder HiFi DNA Assembly Reaction Protocol (New England Biolabs, Frankfurt a. M.). The cDNA of the G proteins was amplified as described above, introducing a *Bam*HI restriction site at the 5′- and a *Hin*dIII at the 3′-end, and cloned into the pFastBac1 backbone via restriction endonuclease reaction protocol. The sequences were verified by sequencing. These pFastBac1 constructs were subsequently used for the generation of recombinant bacmids according to the manufacturer's instructions (Invitrogen).

The H_1R and the G proteins $G\alpha_q$, $G\beta_1$ and $G\gamma_2$ were expressed using the Bac-to-Bac Baculovirus Expression System (Invitrogen). *Spodoptera frugiperda* (*Sf*9) cells were seeded into a 6-well plate (Sarstedt, Nürnbrecht, Germany) at a density of 0.8 x 10^6 cells/well in InsectXpress medium (Lonza, USA) without FCS. The transfection with bacmids was performed as described in manufacturer's instructions (Invitrogen) but using X-tremeGENE™ HP (Roche Diagnostics, Mannheim, Germany) as transfection reagent. After an incubation period of 5 h at 27 °C the transfection mixture was replaced by 2 mL of full growth medium (InsectXpress supplemented with 5% FCS). The P1 baculoviruses were isolated after the *Sf*9 cells were incubated for 72 h at 27 °C, when signs of infection were visible. Amplification of the virus stock was achieved by infecting 30 mL of *Sf*9 cells (2 x 10^6 cells/mL) with 2 mL of P1 and P2 baculoviruses were harvested after 48 h. A further amplification step was performed using 50 mL *Sf*9 cells (1 x 10^6 cells/mL) and 2.5 mL of P2 to obtain high-titre P3 baculoviruses after 48 h of incubation at 27 °C.

To prepare membranes from *Sf*9 cells co-expressing the H_1R + $G\alpha_q$ +$G\beta_1$ +$G\gamma_2$, the cells (50 mL, 1 x 10^6 cells/mL) were co-infected with 2.5 mL of the corresponding P3 virus stocks and incubated for 48 h at 27 °C. Isolation and storage of the membranes as well as the determination of protein concentration was performed as described previously [7,8]. The receptor expression was determined with saturation binding experiments using [^{3}H]mepyramine as radiolabeled tracer as described previously [9] and 0.5 – 1 μg protein/well. The determined $pK_d = 7.93 \pm 0.06$ nM differs slightly from the literature value ($pK_d = 8.35$) [10,11].

[^{35}S]GTPγS binding assay procedure.

The [^{35}S]GTPγS Assay was essentially performed as described previously by Lazewska et al. (2019)[12] with following modifications: The amount of protein was reduced to 1 μg/well and the saponin concentration was decreased to 50 μg/mL. The antagonist mode was performed in the presence of 30 μM histamine.

References

1. Seifert, R.; Wenzel-Seifert, K.; Burckstummer, T.; Pertz, H.H.; Schunack, W.; Dove, S.; Buschauer, A.; Elz, S. Multiple differences in agonist and antagonist pharmacology between human and guinea pig histamine H1-receptor. *J Pharmacol Exp Ther* **2003**, *305*, 1104-1115, doi:10.1124/jpet.103.049619.
2. Baumeister, P.; Erdmann, D.; Biselli, S.; Kagermeier, N.; Elz, S.; Bernhardt, G.; Buschauer, A. [(3) H]UR-DE257: development of a tritium-labeled squaramide-type selective histamine H2 receptor antagonist. *ChemMedChem* **2015**, *10*, 83-93, doi:10.1002/cmdc.201402344.
3. Igel, P. Tritium-Labeled N1-[3-(1H-imidazol-4-yl)propyl]-N2propionylguanidine ([3H]UR-PI294), a High-Affinity Histamine H3 and H4 Receptor Radioligand **2009**.
4. Grätz, L.; Tropmann, K.; Bresinsky, M.; Müller, C.; Bernhardt, G.; Pockes, S. NanoBRET binding assay for histamine H2 receptor ligands using live recombinant HEK293T cells. *Scientific Reports* **2020**, *10*, 13288, doi:10.1038/s41598-020-70332-3.
5. Leurs, R.; Smit, M.J.; Menge, W.M.; Timmerman, H. Pharmacological characterization of the human histamine H2 receptor stably expressed in Chinese hamster ovary cells. *Br J Pharmacol* **1994**, *112*, 847-854, doi:10.1111/j.1476-5381.1994.tb13157.x.
6. Yang, Z.; Han, S.; Keller, M.; Kaiser, A.; Bender, B.J.; Bosse, M.; Burkert, K.; Kogler, L.M.; Wifling, D.; Bernhardt, G., et al. Structural basis of ligand binding modes at the neuropeptide Y Y1 receptor. *Nature* **2018**, *556*, 520-524, doi:10.1038/s41586-018-0046-x.
7. Kelley, M.T.; Burckstummer, T.; Wenzel-Seifert, K.; Dove, S.; Buschauer, A.; Seifert, R. Distinct interaction of human and guinea pig histamine H2-receptor with guanidine-type agonists. *Mol Pharmacol* **2001**, *60*, 1210-1225, doi:10.1124/mol.60.6.1210.
8. Houston, C.; Wenzel-Seifert, K.; Burckstummer, T.; Seifert, R. The human histamine H2-receptor couples more efficiently to Sf9 insect cell Gs-proteins than to insect cell Gq-proteins: limitations of Sf9 cells for the analysis of receptor/Gq-protein coupling. *J Neurochem* **2002**, *80*, 678-696, doi:10.1046/j.0022-3042.2001.00746.x.
9. Bartole, E.; Littmann, T.; Tanaka, M.; Ozawa, T.; Buschauer, A.; Bernhardt, G. [(3)H]UR-DEBa176: A 2,4-Diaminopyrimidine-Type Radioligand Enabling Binding Studies at the Human, Mouse, and Rat Histamine H4 Receptors. *J Med Chem* **2019**, *62*, 8338-8356, doi:10.1021/acs.jmedchem.9b01342.
10. Bosma, R.; Witt, G.; Vaas, L.A.I.; Josimovic, I.; Gribbon, P.; Vischer, H.F.; Gul, S.; Leurs, R. The Target Residence Time of Antihistamines Determines Their Antagonism of the G Protein-Coupled Histamine H1 Receptor. *Front Pharmacol* **2017**, *8*, 667, doi:10.3389/fphar.2017.00667.
11. Strasser, A.; Striegl, B.; Wittmann, H.J.; Seifert, R. Pharmacological profile of histaprodifens at four recombinant histamine H1 receptor species isoforms. *J Pharmacol Exp Ther* **2008**, *324*, 60-71, doi:10.1124/jpet.107.129601.
12. Lazewska, D.; Mogilski, S.; Hagenow, S.; Kuder, K.; Gluch-Lutwin, M.; Siwek, A.; Wiecek, M.; Kaleta, M.; Seibel, U.; Buschauer, A., et al. Alkyl derivatives of 1,3,5-triazine as histamine H4 receptor ligands. *Bioorg Med Chem* **2019**, *27*, 1254-1262, doi:10.1016/j.bmc.2019.02.020.
